# Impact of Hearing Loss Type on Linguistic Development in Children: A Cross-Sectional Study

**DOI:** 10.3390/audiolres14060084

**Published:** 2024-11-27

**Authors:** Nadia Porcar-Gozalbo, Miguel López-Zamora, Beatriz Valles-González, Alejandro Cano-Villagrasa

**Affiliations:** 1 Faculty of Health Sciences, Valencian International University (VIU), 46002 Valencia, Spain; nadia.porcar@professor.universidadviu.com (N.P.-G.); beatriz.valles@professor.universidadviu.com (B.V.-G.); 2 Department of Developmental and Educational Psychology, University of Malaga, 29010 Malaga, Spain; miglopzam@uma.es

**Keywords:** childhood hearing loss, language development, linguistic skills, early intervention, cochlear implants

## Abstract

**Background/Objectives:** Hearing loss in childhood is associated with significant challenges in linguistic and cognitive development, particularly affecting language skills such as syntax, semantics, and pragmatics, which are essential for effective communication and social integration. This study aimed to analyze how different types and degrees of hearing loss impact linguistic development in children, and to identify clinical factors—such as age at diagnosis and years of language intervention—that may predict language performance. **Methods:** This study included a sample of 140 children aged 6 to 12, categorized into seven groups based on their hearing condition: unilateral and bilateral conductive, unilateral and bilateral sensorineural, unilateral and bilateral mixed hearing loss, and a control group with no hearing loss. Linguistic development was assessed using the Clinical Evaluation of Language Fundamentals-5 (CELF-5), a validated tool for diagnosing language disorders. Statistical analyses, including MANOVA and multiple regression, were conducted to evaluate differences in linguistic skills across groups and to determine the predictive value of clinical variables on total language performance. **Results:** The analysis revealed statistically significant differences across groups in all assessed linguistic domains (*p* < 0.001), with children with severe or bilateral hearing loss exhibiting notably lower scores compared to normohearing peers. The multiple regression analysis indicated that type of hearing loss was the strongest predictor of total linguistic performance (β = −0.674), followed by age at diagnosis (β = −0.285) and age of hearing device adaptation (β = −0.220). Years of language intervention also contributed significantly (β = 0.198) but to a lesser extent. **Conclusions:** This study highlights the critical impact of early and comprehensive auditory and language intervention on linguistic outcomes for children with hearing impairments. Early diagnosis and timely adaptation of hearing aids or cochlear implants are essential in mitigating language deficits, particularly in areas like syntax and pragmatic skills. These findings support the need for specialized, long-term interventions tailored to the severity and type of hearing loss to improve language development in this population.

## 1. Introduction

Hearing loss in childhood has significant repercussions on language and cognitive development, directly interfering with essential communication and socialization skills. Early development of oral language, which is crucial for effective socialization and cognitive growth, is particularly vulnerable to disruptions caused by hearing impairment [1]. Numerous studies have emphasized that hearing deficits not only impact speech production but also affect pragmatic skills, vocabulary acquisition, and grammatical abilities, which in turn hinder these children’s capacity to form social relationships and achieve academic success [2]. One of the first limitations resulting from hearing loss is a delay in oral language development, which often leads to difficulties in cognitive tasks such as reasoning, memory, reading comprehension, and problem-solving [3].

Recent studies indicate that children with hearing loss often display atypical developmental patterns at the phonological, morphosyntactic, and lexical-semantic levels, significantly diverging from those observed in their hearing peers [4]. Hearing loss limits a child’s full exposure to the necessary linguistic input for typical language development, affecting not only word acquisition but also the structure and organization of sentences [5]. These challenges are further exacerbated when the child grows up in a family environment lacking adequate stimulation or when early intervention is not provided [6].

The assessment of language difficulties in deaf children is inherently complex due to the multitude of factors involved, such as the degree of hearing loss, the use of cochlear implants, the timing of early intervention, and the characteristics of the communicative environment [7]. One common difficulty among these children is the discrimination of speech sounds, which directly influences their ability to learn and retain new words—especially abstract or less common ones [8]. Research on English- and French-speaking populations has shown that deaf children face notable challenges in understanding functional words (e.g., prepositions and conjunctions), hindering their ability to grasp and produce complex grammatical structures [2,9].

For Spanish-speaking children, studies are more limited, but emerging research has started to explore the distinct aspects of language development in this population. According to recent data, the prevalence of hearing loss in Spain is estimated at approximately 1 to 2 per 1000 newborns, increasing to 5 per 1000 in the child population due to factors acquired during early childhood. For example, Díaz-López and Barajas [10] found that deaf children raised in bilingual sign language environments performed worse on grammatical comprehension tasks compared to hearing children. Although cochlear implants and early rehabilitation have improved outcomes for deaf children, specific limitations remain in the development of complex language processing skills [6].

Cognitive development is also significantly affected by hearing loss, as limited auditory access restricts a child’s ability to comprehend and retain environmental information, which diminishes their curiosity and motivation for learning [11]. A lack of access to enriched information and experiences during early childhood can hinder the acquisition of self-regulation skills, which are crucial for decision-making and self-control in social interactions [12]. Additionally, recent studies suggest that these children may develop more egocentric tendencies or have difficulties with perspective-taking, partly due to limited exposure to conversations and discussions at home, which are less accessible because of hearing loss [13].

The prognosis for children with hearing loss has improved significantly with advances in cochlear implants, providing them with better access to functional oral language [4]. However, challenges persist, particularly in the pragmatic aspects of communication. Children with cochlear implants often have limited exposure to diverse communicative contexts, relying primarily on the language registers of their caregivers or therapists, which can hinder their understanding of social and cultural nuances [14]. Difficulties in interpreting non-verbal cues and social interaction rules also limit their ability to fully participate in social and academic activities [15]. Furthermore, restricted access to a broad range of auditory stimuli during the critical early years of development affects reading comprehension and written expression, skills that depend heavily on a strong foundation in oral language [16,17].

In this context, research has consistently shown that children with hearing impairments face significant challenges across multiple language domains, including phonology, morphosyntax, lexicon, and pragmatics, all of which have repercussions on their academic performance and their ability to interact socially [18]. These findings underscore the need for specialized, individualized interventions to support their development, taking into account factors such as the degree of hearing loss, the timing of intervention, and the characteristics of the family and school environment [19]. Implementing intervention strategies based on cognitive and linguistic development can promote better performance and facilitate greater integration of children with hearing impairments into social and educational environments.

Therefore, the primary objective of this study was to investigate language alterations in children with hearing impairments, focusing on the specific difficulties they face in their linguistic development and the impact on their competencies. To this end, this study assessed sentence and oral text comprehension, as well as the development of linguistic concepts, vocabulary, and morphosyntactic skills, comparing their performance with that of normohearing children. Additionally, this study analyzed the ability to establish semantic connections, follow verbal instructions, and perform specific tasks, as well as the skill to construct and repeat sentences, recognize words in different contexts, and solve tasks related to written language decoding. Pragmatic competencies and the appropriateness of language in communicative situations were also investigated. This approach allowed for the identification of specific areas of difficulty and provided guidance for effective interventions to improve the linguistic development of children with hearing loss.

## 2. Method

### 2.1. Participants

For the present study, a sample of 140 children aged between 8 and 12 years was selected and divided into groups according to their hearing condition. Of these, 76 were boys and 64 were girls, selected through non-probabilistic, intentional sampling in collaboration with reference hospitals in the region. The sample size of 140 participants was determined to ensure sufficient statistical power to detect significant differences between groups. The decision to divide participants into 7 groups of 20 was made with the aim of maintaining a balanced distribution, allowing for detailed comparisons between different types of hearing loss, controlling variables such as age, gender, and socioeconomic status.

To ensure the quality of the sample, a rigorous screening process was implemented for participants incorporated into this study. Initially, all candidates were evaluated through a preliminary questionnaire provided to families, which collected information about medical history, linguistic development, and potential previous diagnoses of neurological or psychiatric problems. This stage helped identify possible exclusion factors and ensure that participants met the established criteria.

Subsequently, a thorough clinical evaluation was conducted to confirm the diagnosis of hearing loss and the level of cognitive development. This included standardized psychometric tests carried out by a team of qualified professionals, who evaluated the IQ of each child to ensure that all had an IQ equal to or greater than 80. The Wechsler Intelligence Scale for Children (WISC-V) was used to measure the cognitive development of each participant, which allowed us to ensure a homogeneous sample and avoid intellectual factors influencing the results of linguistic tests.

Additionally, the participants’ medical histories were reviewed to exclude those with neurological or psychiatric disorders that could interfere with linguistic performance. This review was conducted in collaboration with the reference hospitals, where otolaryngologists and other specialists provided detailed clinical information that helped confirm the children’s eligibility for this study.

This multi-step selection process and the use of various sources of information made it possible to effectively screen the sample, ensuring that only children who strictly met the inclusion criteria were ultimately admitted into this study. The intention was to minimize variability and maximize the validity of the results obtained, contributing to the robustness of the findings and the accuracy of the study conclusions.

The inclusion criteria for this study were as follows: (I) a diagnosis of hearing loss made by a public healthcare institution, ensuring clinical reliability of the diagnosis; and (II) age between 8 and 12 years, a range in which linguistic and cognitive development is particularly sensitive to the effects of hearing loss. Exclusion criteria included (I) a diagnosed neuropsychiatric disorder, such as autism spectrum disorder or any other neurological disorder that could interfere with linguistic performance; and (II) an intelligence quotient below 80, assessed using standardized psychometric tests to ensure a homogeneous sample and to prevent linguistic performance variability due to intellectual factors.

Participants were classified into seven groups based on the type and degree of hearing loss according to their clinical auditory diagnosis. The classification was as follows:

- Group 1 (G1): Unilateral conductive hearing loss (*n* = 20),
- Group 2 (G2): Bilateral conductive hearing loss (*n* = 20),
- Group 3 (G3): Unilateral sensorineural hearing loss (*n* = 20),
- Group 4 (G4): Bilateral sensorineural hearing loss (*n* = 20),
- Group 5 (G5): Unilateral mixed hearing loss (*n* = 20),
- Group 6 (G6): Bilateral mixed hearing loss (*n* = 20),
- Group 7 (G7): Control group composed of normohearing participants without any diagnosis of hearing loss (*n* = 20).

Each participant was diagnosed by the otorhinolaryngology department of their reference hospital, ensuring the accuracy and validity of the classification. The diagnostic procedures included pure-tone audiometry, speech audiometry, and tympanometry. Pure-tone audiometry was used to determine the hearing threshold of each ear and was essential in identifying the degree and type of hearing loss. Speech audiometry assessed the children’s ability to understand speech, providing insight into how hearing loss could impact language perception. Finally, tympanometry evaluated middle ear function, which is relevant for distinguishing conductive hearing loss from other auditory pathologies.

Each family received detailed information about the purpose, procedures, and requirements of this study and signed an informed consent form approving their children’s participation. This consent process met ethical standards for studies involving minors, ensuring parents’ understanding of this study and safeguarding participants’ confidentiality.

### 2.2. Instruments

For evaluating the linguistic development of the children, the Clinical Evaluation of Language Fundamentals-5 (CELF-5) [20] was used, a validated tool for identifying and monitoring language disorders in children and adolescents aged 5 to 15 years. The CELF-5 allows for the assessment of various areas of language, including auditory comprehension, oral expression, morphosyntactic skills, semantics, and pragmatics. In this study, the CELF-5 was administered individually in a 60-min session per participant. The CELF-5 subtests were scored on a dichotomous scale from 0 to 1, where 0 indicated the absence of the assessed skill and 1 its presence. Additionally, a linguistic profile questionnaire with a Likert scale from 0 to 4 was included, where 0 meant “never” and 4 “always”, thus evaluating the frequency and consistency of linguistic skills in everyday contexts.

### 2.3. Procedure

This study received approval from the ethics committee of the University of Málaga (approval code: 120-2023-H). Data collection was conducted at the reference hospitals and in the psychology laboratory at the University, using an acoustically controlled and soundproofed room to avoid distractions and ensure that the results were not influenced by ambient noise. The evaluations were carried out individually, and each session lasted approximately 60 min. Before starting the evaluation session, the procedure was explained again to each child and their family, ensuring their understanding and willingness to participate. During the session, evaluators followed a standardized protocol for administering the CELF-5 to ensure consistency in evaluation. The evaluators were professionals trained in psychology and audiology with experience in administering language assessment tools in children, which guaranteed reliability and validity in data collection.

Data obtained from each CELF-5 subtest were carefully recorded and entered into a protected electronic database to maintain confidentiality and facilitate subsequent statistical analysis. Personal data were anonymized using alphanumeric codes to protect participants’ identities and comply with data confidentiality standards. Each evaluation was supervised and reviewed upon completion to ensure that the information was recorded thoroughly and accurately.

### 2.4. Design

The study design was quasi-experimental and cross-sectional. To analyze the data obtained, the normality of the sample distribution and the homogeneity of variances between groups were first verified using the Shapiro–Wilk test, confirming that the data met the necessary assumptions for applying parametric tests. Descriptive analyses were conducted to characterize the participants and their families sociodemographically, providing general information about the sample in terms of age, gender, type, and degree of hearing loss. This initial description facilitated understanding the sample composition and observing general patterns in the characteristics of the study groups. To assess differences in language skills between hearing-impaired groups and the normohearing group, multivariate analyses of variance (MANOVAs) were performed. This type of analysis enabled simultaneous comparisons of the different language dimensions assessed by the CELF-5 and analyzed whether significant differences existed between groups in each of these areas. MANOVA was chosen because it reduces the risk of Type I error in multiple comparisons and allows for the identification of specific patterns of linguistic difficulty according to the type of hearing loss.

Post hoc analyses were subsequently conducted with Holm–Bonferroni correction to adjust significance levels and reduce the risk of Type I errors in multiple group comparisons. This statistical correction allowed for a more precise evaluation of specific differences between each group and facilitated the identification of the linguistic areas where children with hearing loss experienced greater difficulties compared to the normohearing group. The Holm–Bonferroni correction was chosen for its higher power and sensitivity compared to other corrections, such as the Bonferroni correction. For data processing and statistical analysis, SPSS version 25.0 was used, a tool that allows for advanced multivariate analyses and includes specific procedures for handling complex data. The results of the statistical analysis made it possible to identify patterns of linguistic development impairment across different types of hearing loss, which facilitated the development of specific conclusions and recommendations for support and intervention for this population.

## 3. Results

First, the sociodemographic and clinical characteristics of the 140 participants in the study sample were analyzed. Table 1 provides a detailed description of these characteristics, including sex distribution, degree of hearing loss, age of hearing aid or cochlear implant adaptation, degree of disability, years of language and cognitive intervention, age at diagnosis, and etiology of hearing loss.

As shown in the table, the sample was balanced in terms of gender, with a slightly higher proportion of males (54.3%) compared to females (45.7%). Most participants (49.3%) had mild hearing loss, while 25.7% had moderate loss, and the remaining 25.0% had severe hearing loss. Regarding the age of adaptation of hearing devices (hearing aids or cochlear implants), 59.3% of participants were fitted between the ages of 2 and 4, 25.0% between the ages of 5 and 7, and only 15.7% at age 8 or later. In terms of degree of disability, most participants (66.4%) had a disability level above 66%, while 33.6% had a degree between 33% and 66%. None of the participants had a disability level below 33%. Language and cognitive intervention varied, with the most common intervention period being 3 years (37.1%), followed by interventions lasting 4 years (34.3%), 5 years (15.7%), and 6 years or more (12.9%). Finally, age at diagnosis was early in most cases, with 15.0% diagnosed before the first year of life, 13.6% diagnosed at age two, 12.9% at age three, and 58.6% diagnosed after the age of four. In terms of the etiology of hearing loss, the most common cause was infection (42.9%), followed by congenital malformations (28.6%), prematurity (17.9%), and exposure to ototoxic drugs (10.7%).

The MANOVA conducted to assess differences in linguistic profile measures among G1, G2, G3, G4, G5, G6, and G7 revealed the presence of statistically significant differences (Wilks’ Lambda = 0.003, F(4,135) = 17.723, *p* < 0.001, η^2^*p* = 0.620). As shown in Table 1, significant differences were found in the following variables: Sentence Comprehension, Linguistic Concepts, Morphosyntax, Related Words, Instruction Execution, Phrase Construction, Phrase Repetition, Oral Text Comprehension, Word Comprehension, Word Puzzle, Semantic Relations, and Pragmatic Skills. The results of the ANOVAs related to the linguistic profile are presented in Table 2.

This table provides a detailed comparison of linguistic performance across seven groups (G1 to G7) on various linguistic tasks. The results reveal a consistent pattern across all tasks, with group performance ranking uniformly as G6 < G4 < G5 < G3 < G2 < G1 < G7. Group G7 consistently demonstrates the highest scores, indicating superior linguistic abilities, while G6 shows the lowest scores, reflecting significant deficits. This clear progression highlights substantial differences in linguistic competence across the groups. The mean scores (M) and standard deviations (SD) further emphasize these differences, illustrating a steady decline in performance from G7 to G6. For example, in the Sentence Comprehension task, the mean scores range from 6.95 in G6 to 34.40 in G7, representing a dramatic disparity in ability levels. Similar trends are observed across all tasks, where G7 consistently achieves scores above 33, demonstrating advanced linguistic competence, while G6 remains below 7 in most cases, indicating considerable challenges. Groups in the middle, such as G2 and G3, show moderate performance, often close to each other, suggesting comparable developmental stages. The statistical analysis underscores the robustness of these differences. All tasks exhibit highly significant group differences with *p*-values less than 0.001. For instance, in Sentence Comprehension, the F value is 359.225, confirming the strong statistical differentiation between groups. Moreover, the effect sizes, represented by partial eta-squared (η^2^P), are exceptionally high across all tasks, ranging from 0.928 to 0.944. These values indicate that a substantial proportion of the variance in performance is attributable to group membership. For example, in Sentence Comprehension, η^2^P is 0.942, meaning 94.2% of the variance in scores can be explained by group differences. Morphosyntax, with an η^2^P of 0.944, shows the highest effect size, highlighting the strong impact of group characteristics on linguistic outcomes. The observed effect sizes are not only statistically significant but also clinically meaningful. In behavioral sciences, η^2^P values above 0.14 are considered large, and the values in this study far exceed this threshold. This suggests that the factors differentiating these groups, such as intervention type, severity of hearing loss, or other variables, play a profound role in shaping linguistic performance. The consistency of these findings across all tasks suggests systematic influences that impact all domains of language development. Overall, the disparity in performance across groups underscores the importance of targeted interventions, particularly for lower-performing groups like G6. The advanced performance of G7 highlights the potential outcomes of effective intervention and support, while the challenges faced by G6 emphasize the need for early and individualized assistance. These results provide strong evidence for the importance of clinical and educational efforts tailored to the specific needs of each group to bridge performance gaps and support linguistic development effectively.

Table 3 presents the Pearson correlation coefficients between linguistic variables and clinical factors assessed in this study. These correlations reveal the relationships between specific language skills and variables such as type of hearing loss, age at diagnosis, type of intervention, and rehabilitation duration. The correlation coefficients indicate the strength and direction of these associations, with positive values suggesting a direct relationship (e.g., greater language skill performance with increased years of intervention) and negative values indicating an inverse relationship (e.g., more severe hearing loss associated with lower performance in language skills). Significant correlations (*p* < 0.05) highlight the impact of clinical and demographic factors on various language dimensions, providing insights into how these factors influence linguistic development.

Lastly, a multiple regression analysis was conducted to assess the extent to which the type of hearing loss and other clinical variables predict participants’ total linguistic performance, as measured by the total CELF-5 score. The independent variables included in the model were as follows: type of hearing loss (numerically coded), age at diagnosis, age of hearing aid or cochlear implant (CI) adaptation, and years of language and cognitive intervention. The regression model was statistically significant (F(4, 135) = 102.564, *p* < 0.001), indicating that the selected independent variables explain a substantial proportion of the variance in participants’ total linguistic performance. The adjusted R^2^ of the model was 0.752, suggesting that 75.2% of the variability in total linguistic performance can be explained by the type of hearing loss and the selected clinical variables. Below are the standardized coefficients (β) for each predictor and their respective *p*-values, allowing for evaluation of the relative importance of each independent variable in the model (Table 4).

Analysis of the coefficients shows that the type of hearing loss is the strongest predictor of total linguistic performance (β = −0.674, *p* < 0.001), followed by age at diagnosis (β = −0.285, *p* < 0.001) and age of hearing aid/CI adaptation (β = −0.220, *p* < 0.001). The variable for years of language and cognitive intervention also has a significant effect on linguistic performance (β = 0.198, *p* < 0.001), though its contribution is smaller compared to the other predictors.

## 4. Discussion

The primary objective of this study was to examine the linguistic differences among children with varying types and degrees of hearing loss in comparison to their normohearing peers. By evaluating core language skills—including sentence comprehension, morphosyntax, pragmatic skills, and semantic relations—this study sought to pinpoint the specific linguistic challenges encountered by children with hearing impairments and assess the impact of clinical factors such as age of diagnosis, age of hearing aid or cochlear implant adaptation, and years of language intervention on their linguistic performance. The findings from this research validate the initial hypotheses: children with hearing impairments, particularly those with bilateral and severe forms, exhibit significantly lower linguistic outcomes across all measured domains compared to their normohearing peers. Additionally, the data indicate that earlier diagnosis, younger age at device adaptation, and prolonged language intervention correlate with better linguistic outcomes, underscoring the importance of early intervention and sustained support.

### 4.1. Impact on Language Domains

These findings offer a nuanced understanding of the linguistic obstacles faced by children with hearing impairments, reinforcing the critical role of consistent and high-quality auditory input for language development. The statistically significant differences observed across all linguistic domains highlight the comprehensive impact of hearing loss on language acquisition, affecting not only vocabulary but also complex linguistic areas such as syntax, pragmatics, and sentence processing. These results echo a common theme in auditory and linguistic research: the timing and consistency of auditory input are crucial in shaping language outcomes in children with hearing impairments. This finding aligns with existing research suggesting that auditory input functions as a foundational scaffold for language skills, with disruptions to this input causing cascading effects on both linguistic and cognitive development [21,22].

### 4.2. Morphosyntax and Pragmatics

One of the most notable observations from this study is the gradient of linguistic performance that correlates with the severity and type of hearing impairment, particularly in areas like morphosyntax and pragmatics. These areas are among the most cognitively demanding, requiring children to process multiple language cues simultaneously—a task that becomes significantly more challenging in the absence of complete auditory input [23,24,25,26]. Children with severe or bilateral hearing impairments, who performed lower in morphosyntactic skills, illustrate how hearing loss can interrupt the natural acquisition of grammatical structures. This disruption likely arises from a reduced ability to detect phonetic cues and distinguish word boundaries, a phenomenon supported by Svirsky et al., who found that even children with cochlear implants often lagged in syntax comprehension due to the lack of early exposure to nuanced sound cues essential for processing grammar [25]. The large effect sizes observed for morphosyntactic skills in this study emphasize that syntax is particularly vulnerable to auditory deprivation, underscoring the unique challenges in narrowing linguistic gaps in this domain.

### 4.3. Pragmatic and Social Aspects of Language

The pragmatic and social aspects of language add further complexity for children with hearing impairments. Pragmatic skills, which include understanding social cues, conversational turn-taking, and interpreting the subtle aspects of language that convey social context, are particularly difficult to acquire without incidental learning through auditory exposure. Botting and Conti-Ramsden underscore that pragmatic language development relies heavily on observing and participating in social interactions—opportunities that are often limited for children with hearing loss due to communication barriers [26]. Consequently, these children frequently miss out on overheard conversations and incidental learning moments, which significantly contribute to pragmatic language development in normohearing children [27,28,29,30]. The deficits in pragmatic skills observed in this study suggest that children with hearing impairments may develop more rigid conversational styles and struggle with adapting their language spontaneously, a finding that aligns with Ketelaar et al., who noted that pragmatic language skills are not only linguistically based but also connected to cognitive and socio-emotional factors, which are indirectly impacted by hearing loss [31]. These findings highlight the need for intervention programs that specifically address pragmatic skills, as traditional language instruction may overlook these socially embedded aspects of language.

### 4.4. Semantic Relations and Vocabulary

In terms of semantic relations and vocabulary breadth, these areas emerged as critical challenges for children with hearing impairments. Unlike basic language skills, which can sometimes be compensated for through explicit teaching, semantic networks and lexical connections require continuous and varied language exposure over time. The limited vocabulary breadth and depth observed in children with hearing impairments in this study is consistent with findings by Spencer and Marschark, who noted that these children typically acquire words at a slower pace and have a more restricted vocabulary, likely due to fewer word-learning opportunities [28]. Additionally, the observed limitations in semantic relations in this study indicate that children with hearing impairments may struggle to form connections between words and concepts, impacting not only their language abilities but also their performance in academic subjects requiring conceptual understanding. This supports the theory that vocabulary depth—understanding words across different contexts—is as critical as vocabulary breadth, highlighting the need for targeted interventions that promote both [27,32].

### 4.5. Clinical Factors and Language Outcomes

The correlations between clinical factors—such as age at diagnosis, age of hearing device adaptation, and years of intervention—and linguistic outcomes reinforce the importance of early diagnosis and long-term support. The positive association between early diagnosis and improved language outcomes underscores a critical window for language acquisition during early childhood, a period when the brain’s plasticity is heightened and most receptive to language input [29]. Delayed diagnosis or late adaptation of hearing devices may reduce the effectiveness of this developmental window, making it more difficult for children to catch up linguistically. This finding is consistent with studies by Tomblin et al., who showed that early intervention can significantly influence language trajectories by optimizing auditory and language input during crucial developmental stages [3]. However, even with timely intervention, it is evident that severe and bilateral hearing impairments continue to present challenges that require tailored support. The positive correlation between years of intervention and linguistic improvement found in this study suggests that consistent support can mitigate some of these challenges over time but may not fully address the gaps in complex linguistic areas like pragmatics and syntax.

### 4.6. Recommendations for Intervention

Moreover, the findings of this study highlight a pressing need for specialized intervention approaches that go beyond general language instruction. Conventional intervention programs often emphasize basic language acquisition, focusing primarily on vocabulary and sentence structure, but may overlook the specific needs associated with pragmatic, semantic, and morphosyntactic skills. To address these gaps, interventions should incorporate social language training, pragmatic exercises, and syntax-focused activities that are frequently absent in standard language programs. Niparko et al. emphasize that hearing devices alone cannot compensate for the lack of incidental learning and the subtle nuances of social language, suggesting that interventions should also focus on social skills development [29]. Additionally, incorporating multimodal strategies that leverage visual, auditory, and contextual cues may enhance comprehension and retention, especially for abstract linguistic concepts. This is particularly relevant for semantic learning, where forming connections between words and concepts requires exposure to a variety of contexts—something that children with hearing impairments may benefit from in a structured, multimodal intervention setting [33,34].

### 4.7. Limitations and Future Directions

Reflecting on these findings, this study underscores that early diagnosis, consistent intervention, and timely adaptation of hearing aids or cochlear implants are crucial for optimizing language outcomes in children with hearing impairments. The results reinforce the understanding that both the quality and quantity of auditory input play a critical role in language acquisition, particularly for linguistic skills that depend on complex language structures and social nuances. In line with previous research, this study affirms that targeted, individualized language interventions—initiated as early as possible—can significantly enhance linguistic development and reduce disparities between children with hearing impairments and their normohearing peers.

While this study provides important insights, several limitations should be noted. The sample was limited to a specific age range and included only children diagnosed and treated within particular clinical parameters, which may affect the generalizability of the findings to broader populations. Additionally, this study did not account for variations in intervention quality or the specific language exposure each child received, both of which could influence language outcomes. Future research could address these limitations by incorporating a larger and more diverse sample, evaluating the impact of various types of intervention, and examining the role of the home language environment.

Looking ahead, longitudinal studies could offer a more comprehensive view of the long-term effects of hearing loss on language development and the effectiveness of different interventions over time. Future research should also investigate the impact of advancements in hearing technology and educational support programs on linguistic outcomes, as these developments have the potential to further bridge the gap in language skills between children with hearing impairments and their normohearing peers.

## 5. Conclusions

This study provides significant insights into the specific linguistic challenges faced by children with varying degrees and types of hearing impairments, highlighting the importance of early diagnosis, timely adaptation of hearing devices, and sustained language intervention in improving language outcomes. The findings confirm that children with severe or bilateral hearing loss exhibit notable deficits in a range of language domains, including morphosyntax, sentence comprehension, pragmatics, and semantic relations, compared to their normohearing peers. These results underscore the complex impact of hearing loss on language acquisition, affecting not only vocabulary and basic comprehension but also the more intricate aspects of language that are essential for effective communication and social interaction.

One of the key takeaways from this study is the critical role of early and consistent auditory input in shaping linguistic development. Children diagnosed at younger ages and fitted with hearing devices earlier showed better linguistic performance, reinforcing the importance of leveraging sensitive developmental windows for language acquisition. This study’s findings suggest that while hearing devices and interventions can improve language outcomes, they may not fully bridge the linguistic gap in complex areas like syntax and pragmatics. This underscores the need for comprehensive and individualized intervention approaches that extend beyond conventional language instruction to include social language training, pragmatic exercises, and support for advanced language structures.

Moreover, the significant correlations between clinical factors (such as age of diagnosis, age of device adaptation, and years of intervention) and linguistic performance highlight the value of ongoing and tailored support for children with hearing impairments. Consistent with the existing literature, this study reinforces the notion that language development in children with hearing loss benefits from multifaceted interventions that are both timely and adaptable to individual needs.

In conclusion, this study affirms the importance of structured, early, and sustained interventions in supporting linguistic growth among children with hearing impairments. While advancements in hearing technology and intervention strategies continue to offer promising pathways, ongoing research is essential to identify the most effective approaches for addressing complex language deficits and fostering linguistic parity between children with hearing impairments and their normohearing peers. These findings call for continued efforts to improve early diagnosis, access to high-quality auditory input, and specialized language support, which together hold the potential to significantly enhance language outcomes and quality of life for children with hearing impairments.

## Figures and Tables

**Table 1:** Characterization of study participants.

	N	Percentage
Sex		
Male	76	51.6
Female	64	48.4
Degree of Hearing Loss		
Mild	69	33.3
Moderate	36	33.4
Severe	15	33.3
Age of Hearing Aid or Cochlear Implant Adaptation		
2–4 years	83	21.7
5–7 years	35	56.6
8–10 years	2	21.7
Degree of Disability		
Less than 33%	0	0
Between 33% and 66%	47	78.3
More than 66%	13	21.7
Years of Language and Cognitive Intervention		
3 years	52	86.7
4 years	4	6.7
5 years	2	3.3
6 years or more	2	3.3
Age at Diagnosis		
1 year or less	21	35
2 years	19	31.7
3 years	18	30
4 years or more	2	3.3

**Table 2:** ANOVA results for linguistic profile.

Profile Linguistic	G1 (n = 20)		G2 (n = 20)		G3 (n = 20)		G4 (n = 20)		G5 (n = 20)		G6 (n = 20)		G7 (n = 20)		F(4,135)	η^2^*p*	Differences Between Groups
	M	SD	M	SD	M	SD	M	SD	M	SD	M	SD	M	SD			
Sentence Comprehension	25.40	1.759	22.50	2.236	19.85	2.346	9.55	2.481	15.45	2.212	6.95	2.212	34.40	2.326	359.225 *	0.942	G6 < G4 < G5 < G3 < G2 < G1 < G7
Linguistic Concepts	24.40	1.875	22.45	2.564	18.85	2.277	10.05	2.373	15.65	2.323	7.05	2.064	33.25	2.124	317.972 *	0.935	G6 < G4 < G5 < G3 < G2 < G1 < G7
Morphosyntax	25.85	1.927	22.40	2.371	18.30	2.598	11.10	2.360	14.80	2.067	7.35	2.134	35.20	1.704	375.057 *	0.944	G6 < G4 < G5 < G3 < G2 < G1 < G7
Related Words	25.35	1.663	22.65	2.498	18.80	2.567	11.45	2.305	16.00	2.224	6.35	1.755	33.80	2.648	323.555 *	0.936	G6 < G4 < G5 < G3 < G2 < G1 < G7
Instruction Execution	25.45	1.731	22.65	2.368	18.90	2.673	10.80	2.505	15.90	2.075	6.35	2.033	34.60	2.137	356.171 *	0.941	G6 < G4 < G5 < G3 < G2 < G1 < G7
Phrase Construction	24.25	2.173	22.65	2.560	19.20	3.205	11.25	2.573	14.90	2.049	7.50	2.115	35.05	1.905	292.397 *	0.930	G6 < G4 < G5 < G3 < G2 < G1 < G7
Phrase Repetition	25.05	2.188	22.80	2.215	19.25	2.673	10.00	2.616	15.75	2.197	6.90	2.075	35.65	2.581	334.260 *	0.938	G6 < G4 < G5 < G3 < G2 < G1 < G7
Oral Text Comprehension	24.75	1.943	22.25	2.221	19.85	2.961	11.10	3.024	16.20	2.067	6.60	2.010	35.25	1.773	325.414 *	0.936	G6 < G4 < G5 < G3 < G2 < G1 < G7
Word Comprehension	25.85	2.134	21.90	2.024	18.45	2.373	11.05	2.605	16.00	2.384	6.65	1.981	34.60	2.280	340.434 *	0.939	G6 < G4 < G5 < G3 < G2 < G1 < G7
Word Puzzle	24.60	2.088	22.20	2.441	19.25	2.971	9.90	2.269	15.50	2.351	7.30	2.227	34.35	2.560	287.483 *	0.928	G6 < G4 < G5 < G3 < G2 < G1 < G7
Semantic Relations	25.10	1.944	23.00	2.596	18.30	2.677	11.35	2.434	15.70	1.949	6.75	2.173	34.15	2.300	313.265 *	0.934	G6 < G4 < G5 < G3 < G2 < G1 < G7
Pragmatic Skills	24.55	2.164	22.60	2.458	18.50	2.856	10.55	2.625	14.95	2.305	7.25	1.860	35.00	2.384	304.984 *	0.932	G6 < G4 < G5 < G3 < G2 < G1 < G7

* *p* < 0.05.

**Table 3:** Pearson correlation results between linguistic variables.

	Sentence Comprehension	Linguistic Concepts	Morphosyntax	Related Words	Type of Hearing Loss	Age at Diagnosis	Type of Intervention	Rehabilitation Duration
Sentence Comprehension	1							
Linguistic Concepts	0.912 *	1						
Morphosyntax	0.901 *	0.935 *	1					
Related Words	0.883 *	0.917 *	0.926 *	1				
Type of Hearing Loss	−0.934 *	−0.912 *	−0.923 *	−0.901 *	1			
Age at Diagnosis	−0.870 *	−0.845 *	−0.860 *	−0.840 *	0.920 *	1		
Type of Intervention	0.810 *	0.795 *	0.805 *	0.800 *	−0.850 *	−0.895 *	1	
Rehabilitation Duration	0.825 *	0.812 *	0.828 *	0.835 *	−0.880 *	−0.875 *	0.905 *	1

* *p* < 0.05.

**Table 4:** Multiple regression analysis for linguistic performance.

	β	Standard Error	t	*p*-Value
Type of Hearing Loss	−0.674	0.048	−14.042	<0.001
Age at Diagnosis	−0.285	0.051	−5.588	<0.001
Age of Hearing Aid/CI Adaptation	−0.220	0.050	−4.400	<0.001
Years of Language/Cognition Intervention	0.198	0.047	4.213	<0.001

## Data Availability

The original data presented in this study are openly available in FigShareat https://doi.org/10.6084/m9.figshare.27647532.v1.
